# Regulatory Non-coding RNAs Network in Non-alcoholic Fatty Liver Disease

**DOI:** 10.3389/fphys.2019.00279

**Published:** 2019-03-19

**Authors:** Siti Aishah Sulaiman, Nor I. A. Muhsin, Rahman Jamal

**Affiliations:** UKM Medical Molecular Biology Institute, Universiti Kebangsaan Malaysia, Kuala Lumpur, Malaysia

**Keywords:** lncRNAs, circRNAs, microRNAs, NAFLD, NASH, biomarker

## Abstract

Non-alcoholic fatty liver disease (NAFLD) spectrum comprises simple steatosis and non-alcoholic steatohepatitis (NASH) that can lead to fibrosis and cirrhosis. The patients usually have no history of excessive alcohol consumption and other etiologies that can cause fatty liver. Understanding of the pathophysiology of NAFLD has revealed that non-coding RNAs (ncRNAs) play significant roles in modulating the disease susceptibility, pathogenesis and progression. Currently, the ncRNAs are grouped according to their sizes and their regulatory or housekeeping functions. Each of these ncRNAs has a wide range of involvement in the regulation of the genes and biological pathways. Here, we briefly review the current literature the regulatory ncRNAs in NAFLD pathogenesis and progression, mainly the microRNAs, long non-coding RNAs and circular RNAs. We also discuss the co-regulatory functions and interactions between these ncRNAs in modulating the disease pathogenesis. Elucidation of ncRNAs in NAFLD may facilitate the identification of early diagnostic biomarkers and development of therapeutic strategies for NAFLD.

## Introduction

Non-alcoholic fatty liver disease (NAFLD) is one of the common chronic liver diseases. It is characterized by the presence of hepatic steatosis (>5–10% of fatty hepatocytes) in an individual without a history of excessive alcohol consumption and other disease etiologies that can cause fatty liver (Araújo et al., 2018). Currently, NAFLD prevalence in the general population is about 24–25% (Bedogni et al., 2005; Younossi et al., 2016, 2017), with the highest prevalence reported in South America and the Middle East, followed by Asia, United States, and Europe (Younossi et al., 2016). In parallel with the increasing incidence of obesity and diabetes, NAFLD will be the leading cause of cirrhosis and HCC in the next 5 years, surpassing hepatitis infection (Streba et al., 2015). Therefore, research on NAFLD pathogenesis, early diagnosis, and biomarkers as well as identification of the therapeutic targets for NAFLD are necessary to reduce the burden of this disease.

Non-alcoholic fatty liver disease is a spectrum disease, in which untreated patients with liver steatosis or non-alcoholic fatty liver (NAFL) can progress to non-alcoholic steatohepatitis (NASH) that further increases the risk of developing cirrhosis and hepatocellular carcinoma (HCC) (Streba et al., 2015). Similar to other metabolic diseases, NAFLD is a multi-factorial disease in which the genetic predisposition, environmental exposures and lifestyle factors can modulate susceptibility to the disease and progression (Wruck et al., 2017). Previous studies have shown that non-coding RNAs (ncRNAs) are implicated in the etiology of NAFLD and possibly be the key mediators in its pathogenesis (Jiang and Zhang, 2017; Lin et al., 2017; Hanson et al., 2018; Turchinovich et al., 2018). These ncRNAs are constitutively expressed and can regulate biological processes, genes and proteins (Yamamura et al., 2018). In this mini-review, we aimed to discuss the role of ncRNAs in NAFLD development and progression, as well as the co-regulatory interaction between these ncRNAs.

## Pathogenesis: Fatty Liver Progression to NASH and Fibrosis

Fatty liver is defined by the accumulation of the triglycerides (TGs) in hepatocytes, due to an imbalance between energy consumption [free fatty acids (FFAs) uptake and *de novo* lipogenesis] and energy metabolism (fatty acid oxidation, lipoprotein packaging and transport) (Peverill et al., 2014; Manne et al., 2018). For most of the patients, this fatty liver condition is non-pathogenic and can be reversed by appropriate interventions, though about 20–30% of them will progress to NASH (Younossi et al., 2017). The exact mechanisms of how fatty liver progresses to NASH is not fully understood. Oxidative stress and inflammation induced by lipotoxicity appear to be the key mechanism in NASH progression (Peverill et al., 2014; Manne et al., 2018). A “two-hit” hypothesis proposes that NASH progression starts with the first hit of insulin resistance that contributes to hepatic steatosis (Gentile and Pagliassotti, 2008) and the second hit of the inflammatory cytokines induced by the oxidative stress (Sumida et al., 2013). However, recent findings have shown that NASH progression is far more complex and the “two-hit” hypothesis is not sufficient to describe the pathogenesis. The interactions and cross-talks between the liver parenchymal (hepatocytes) and non-parenchymal cells [hepatic stellate cells (HSCs)], Kupffer cells (stellate macrophages), and various immune cells are recently recognized to participate in NASH progression (Magee et al., 2016). Excessive TGs accumulation promotes the hepatocellular injury (ballooning) which will stimulate the inflammatory response and activates the liver immune cells and HSCs (Magee et al., 2016). In response to hepatocyte injury, HSCs transform or activate into a myofibroblast phenotype and promotes the secretion of cytokines and components of the extracellular matrix (ECM) to protect the liver (Magee et al., 2016). Therefore, in the setting of chronic inflammation, prolonged secretion of ECM components results in hepatic scarring and fibrosis (Friedman, 2008). Due to these complicated interactions, a “multiple-hit” hypothesis is more acceptable to describe NASH progression (Tilg and Moschen, 2010). In this “multiple-hit” model, several dysregulated pathways and insults can act in parallel in an individual in combination with genetic predisposition (Tilg and Moschen, 2010), thus increases the risk of developing NASH. Due to the word limit imposed, this review will not discuss NAFLD/NASH pathogenesis in details, as previous reviews have covered the topic extensively, including the various mechanisms, pathways and genetic factors involved (Tilg and Moschen, 2010; Sumida et al., 2013; Peverill et al., 2014; Magee et al., 2016; Ananthanarayanan, 2018; Eslam et al., 2018; Ibrahim et al., 2018; Manne et al., 2018). Despite the fact that NASH patients have a greater risk of developing end-stage liver diseases (Streba et al., 2015), it is unclear why some NAFL patients progress to NASH while some others do not.

## Non-Coding RNAs in NAFLD

Non-coding RNAs (ncRNAs) refers to a group of RNAs that do not encode for a protein, and most of these ncRNAs are the products of alternative splicing with the larger transcripts become the precursors for smaller ncRNAs (Djebali et al., 2012). Initially, these ncRNAs are considered to be the genome “junks,” but in recent years, ncRNAs are shown to be involved in various cellular processes and disease stages, with emerging evidence of their interactions with each other to form a complex regulatory network (Yamamura et al., 2018). In general, ncRNAs belong into two groups according to their lengths, i.e., the small ncRNAs (<200 nucleotides) and the long ncRNAs (>200 nucleotides) (Djebali et al., 2012). Within these two groups, they are further characterized according to their functions. Among the small ncRNAs, the transfer RNAs, small nucleolar RNAs and small nuclear RNAs are known as the small housekeeping ncRNAs, whereas PIWI-interacting RNAs (piRNAs), circular (circRNAs) and microRNAs (miRNAs) are known as the small regulatory ncRNAs (Djebali et al., 2012). Similarly, for the long ncRNAs, the ribosomal RNA is the housekeeping long ncRNA, whereas the long ncRNAs (lncRNAs) and enhancer RNAs (eRNAs) are known as the long regulatory ncRNAs (Djebali et al., 2012; Hon et al., 2017).

### MicroRNAs (miRNAs)

MicroRNAs (miRNAs) are highly conserved short single-stranded ncRNAs (∼18–22 nucleotides) that can regulate gene expression via specific complementary binding to target mRNA, and results in either mRNA degradation (perfect binding) or translational suppression (imperfect binding), though this silencing of mRNA expression can be reversed (Vishnoi and Rani, 2017). MiRNAs are the most studied ncRNAs with their biogenesis and processing are well-defined (O’Brien et al., 2018). Majority of the miRNAs are produced via the canonical pathway that starts with miRNA transcription step thus producing long primary transcripts (pri-miRNAs) (O’Brien et al., 2018). The pri-miRNAs are then processed by RNase III-type enzyme, Drosha to generate the hairpin precursors (pre-miRNAs). These pre-miRNAs are then exported to the cytoplasm, where they are further processed into mature miRNA duplex by the RNase III protein Dicer (O’Brien et al., 2018). Finally, one of the miRNA duplex strands is incorporated into the RNA-induced silencing complex (RISC), to exert their regulatory function (O’Brien et al., 2018). A previous review has extensively discussed miRNA biogenesis and their regulatory mechanism in details, including the non-canonical biogenesis pathway and miRNA modifications (O’Brien et al., 2018), therefore these topics will not be discussed here. As for NAFLD/NASH, miRNAs roles have been reviewed in terms of the disease pathogenesis, as a biomarker and as a potential therapeutic target (Baffy, 2015; Gerhard and DiStefano, 2015; DiStefano and Gerhard, 2016; He et al., 2016; Otsuka et al., 2016; Lin et al., 2017). Hence, this review will only highlight those findings. All the miRNAs identified in circulating samples of the NAFLD/NASH patients are summarized in Table 1.

**Table 1:** Reported expression of microRNAs identified in NAFLD, NASH and fibrosis patients.

Non-coding RNAs	Liver Disease	Tissue Expression	Circulating Expression
miR-122	NAFLD	Reduced (Latorre et al., 2017)	Increased (Cermelli et al., 2011; Yamada et al., 2013; Tan et al., 2014; Pirola et al., 2015; Liu et al., 2016; Salvoza et al., 2016; Brandt et al., 2018)
	NASH	Reduced (Cheung et al., 2008; Pirola et al., 2015; Aitana et al., 2016)	Increased (Becker et al., 2015; Pirola et al., 2015; Liu et al., 2016)
	Fibrosis	Reduced (Miyaaki et al., 2014)	Reduced (Miyaaki et al., 2014; Akuta et al., 2016)
miR-34a	NAFLD	NA	Increased (Cermelli et al., 2011; Yamada et al., 2013; Salvoza et al., 2016)
	NASH	Increased (Cheung et al., 2008; Aitana et al., 2016)	Increased (Liu et al., 2016)
miR-33a/b	NASH	Increased (Joel et al., 2016)	NA
miR-103/miR-107	NAFLD	NA	Increased (Xu Q. et al., 2015)
miR-21	NAFLD	NA	Increased (Yamada et al., 2013; Liu et al., 2016)
	NASH	NA	Increased (Becker et al., 2015)
miR-192	NAFLD	NA	Increased (Tan et al., 2014; Pirola et al., 2015; Liu et al., 2016)
	NASH	Reduced (Pirola et al., 2015)	Increased (Becker et al., 2015; Pirola et al., 2015; Liu et al., 2016)
miR-223	NAFLD	Reduced	Increased
	NASH	Reduced	Increased (Becker et al., 2015)
miR-29c	NASH	Reduced (Aitana et al., 2016)	NA
miR-181d, miR-99a, miR-197	NASH	NA	Reduced (Celikbilek et al., 2014)
miR-16	NASH	NA	Increased (Cermelli et al., 2011; Liu et al., 2016)
miR-146b	NAFLD	Reduced (Latorre et al., 2017)	Increased (Liu et al., 2016)
	NASH	Increased (Cheung et al., 2008)	Reduced (Celikbilek et al., 2014)
miR-19a, miR-19b, miR-125, miR-375	NAFLD	Reduced	Increased (Pirola et al., 2015)
	NASH	Reduced	Increased (Pirola et al., 2015)
miR-1290, miR-27b, miR-148a	NAFLD	NA	Increased (Tan et al., 2014)
miR-99a	NAFLD	NA	Increased (Tan et al., 2014)
	NASH	NA	Reduced (Celikbilek et al., 2014)
miR-139, miR-30b, miR-442	NAFLD	Reduced (Latorre et al., 2017)	NA
miR-451	NAFLD	NA	Increased (Yamada et al., 2013)


#### MicroRNAs in Hepatic Lipid Regulation, Steatosis and NASH

**FIGURE 1:**
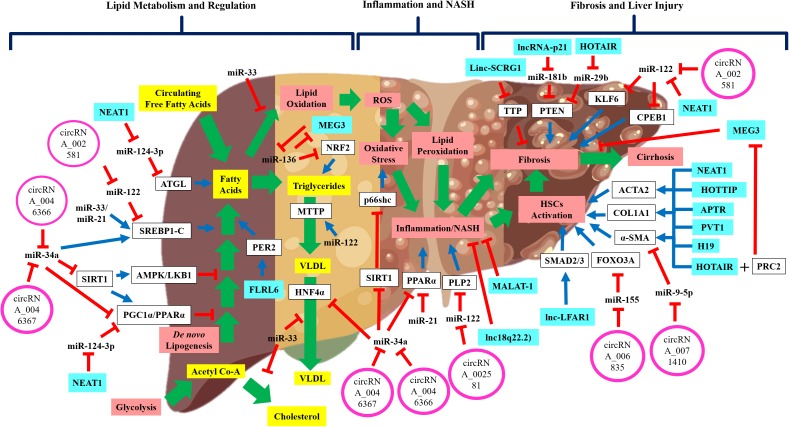
The schematic diagram shows the regulatory non-coding RNAs in NAFLD disease development and progression. Wide-range of pathways and processes are regulated by non-coding RNAs in NAFLD development and progression. Emerging evidence shows that these regulatory non-coding RNAs interact with each other thus implying the complex regulation of NAFLD development and progression. Green arrow: pathway progression, Blue arrow: positive regulation, red inhibition arrow: negative regulation, and plus sign: combined action.

The most well-known hepatic miRNAs is miR-122 that is highly expressed in the liver, which acts as a beacon for hepatocyte status (Cheung et al., 2008; Hsu et al., 2012; Csak et al., 2015; Pirola et al., 2015) and to preserve adult liver signature profile (Krützfeldt et al., 2005) (Figure 1). Transient inhibition of miR-122 expression resulted in a lower cholesterol level, due to increased hepatic fatty acid oxidation (Krützfeldt et al., 2005; Esau et al., 2006; Elmén et al., 2008), and improvement in hepatic steatosis (Esau et al., 2006). However, total knockout of miR-122 expression was detrimental, as it caused higher TGs accumulation and hepatic micro-steatosis that progressed to NASH and fibrosis (Hsu et al., 2012). Consistent with these findings, reduced expression of miR-122 was observed in hepatic tissues of NAFLD and NASH, both in animal models (Alisi et al., 2010; Tryndyak et al., 2012) and human patients (Cheung et al., 2008; Pirola et al., 2015; Aitana et al., 2016; Latorre et al., 2017).

Besides miR-122, several other miRNAs are involved in NAFLD pathogenesis, particularly in liver steatosis (Table 1 and Figure 1). One is miR-34a, which was shown to be up-regulated in both tissue and serum samples of NAFLD/NASH patients (Cheung et al., 2008; Yamada et al., 2013; Salvoza et al., 2016). miR-34a was shown to downregulate expression of several key genes in NAFLD pathogenesis, including *HNF4α* (Xu Y. et al., 2015), *PPARα* and *SIRT1* expressions (Ding et al., 2015; Shan et al., 2015). Dysregulation of these genes has been associated with higher TGs accumulation and liver steatosis (Ding et al., 2015; Shan et al., 2015; Xu Y. et al., 2015). Furthermore, this reduction of *SIRT1* expression also initiated the activation of pro-apoptotic genes, including *P66SHC* (Shan et al., 2015) and *P53* (Chang et al., 2007; Castro et al., 2013), which could increase hepatocyte susceptibility toward oxidative stress and apoptosis. Another miRNA involved in NAFLD is miR-21, which was up-regulated in the serum of NASH patients (Becker et al., 2015), but downregulation of miR-21 expression resulted in reduced hepatic inflammation (Loyer et al., 2016). It is important to note that miR-21 is mainly expressed by the inflammatory and biliary cells, not hepatocytes (Loyer et al., 2016), thus the exact mechanism of how miR-21 is involved in NASH remained unknown. Previous studies also have shown that miR-33 family members are the critical regulators of lipid metabolism and transport (Rayner et al., 2010; Dávalos et al., 2011) and their expressions were increased in NASH patients (Joel et al., 2016). A recent exploratory study using the aerobic exercise training as a therapy to reduce lipid levels in mice, had shown that the lipid-lowering effects observed were through miR-33 dependent autophagy (Ghareghani et al., 2018), thus confirming the regulatory role of miR-33 in lipid metabolism.

#### MicroRNAs in Hepatic Fibrosis and Cirrhosis

Hepatocyte-specific miR-122 expression was shown to be elevated in the serum or blood of the animal models of liver damage (Tryndyak et al., 2012; Clarke et al., 2014; Pirola et al., 2015; Yamada et al., 2015) and human NAFLD patients (Cermelli et al., 2011; Yamada et al., 2013; Celikbilek et al., 2014; Ward et al., 2014; Becker et al., 2015; Akuta et al., 2016; Salvoza et al., 2016). In contrast, the expression of miR-122 was found to be reduced in the liver tissues of NAFLD animal models and NAFLD patients (Cheung et al., 2008; Alisi et al., 2010; Tryndyak et al., 2012; Pirola et al., 2015; Aitana et al., 2016; Latorre et al., 2017). The reason for this discrepancy might be due to miR-122 is secreted out from damaged hepatocytes, and this was supported by the evidence of miR-122 localization that was mainly at the boundary of cell wall rather than in the cytoplasm (Su et al., 2018). As miR-122 is exported out regularly into the circulation, miR-122 possesses vast potential as a biomarker for liver injury and fibrosis progression.

Besides miR-122, inhibition of miR-21 resulted in reduced liver fibrosis and since miR-21 is mainly expressed by the inflammatory and biliary cells (Loyer et al., 2016), thus miR-21 may play a significant role in cross-talk between liver microenvironment and hepatocytes, though further study is needed to determine this relationship. On the other hand, overexpression of miR-29b was also shown to inhibit liver fibrosis via *COLIA1* and collagens in HSCs (Ogawa et al., 2012). In fact, the expression of the whole miR-29 family was down-regulated in mice with liver fibrosis (Roy et al., 2015) and miR-29c expression was reduced in the liver of NASH patients (Aitana et al., 2016). Closer inspection on these molecules revealed that miR-29b is the leading player of hepatic fibrosis, where it could regulate several pro-fibrotic genes, including *COLIA1*, *SHH*, and *cMYC* genes and the introduction of miR-29b managed to reduce collagen disposition with improvements in liver health (Kumar et al., 2016). Although these continuous updates on miRNAs involvement in NAFLD/NASH are enlightening, the practicality of using these miRNAs as therapeutic targets requires further assessments. The reason is that many of these miRNAs regulate multiple targets simultaneously and thus may unintendedly affect other important pathways. Therefore, most current studies on miRNAs are moving forward into looking at the efficacy, pharmacokinetics and pharmacodynamics of miRNA-based therapy to stop and cure the disease progression (Chakraborty et al., 2017).

### Long Non-coding RNA (lncRNAs)

Long non-coding RNAs (lncRNAs) are RNA transcripts (>200 nucleotides in size) without the protein translation capacity and accounted for the majority of ncRNAs (Quinn and Chang, 2015). LncRNAs are transcribed from intergenic, exonic or the distal protein-coding regions by the RNA polymerase II and capped at 5′-end and polyadenylated at 3′-end (Quinn and Chang, 2015). Due to their locations are mainly in the poorly conserved regions of the genome, lncRNAs are difficult to characterize and highly diverse in their lengths, localizations and functions (Ma et al., 2013; Quinn and Chang, 2015). Furthermore, they can form secondary and tertiary structures to interact with other molecules (Dhanoa et al., 2018), thus adding more to their heterogeneity and diversity. Generally, lncRNAs are grouped based on their structure, function, localization and interaction with other molecules (Dhanoa et al., 2018). Currently, there are five classes of lncRNAs: (1) sense lncRNAs, (2) antisense lncRNAs, (3) bidirectional lncRNAs, (4) intronic lncRNAs and (5) intergenic lncRNAs (Dhanoa et al., 2018). In terms of their functions, there are a few that have been identified: (1) as a decoy that binds a targeted protein or miRNA to suppress their functions, (2) as a guide by binding to the proteins and directs their localization, (3) as a scaffold by acting as a platform for the intermolecular interactions between proteins and RNAs, and (4) as a signal by interacting with transcription factors or chromatin-modifying enzymes to regulate gene expression (Ma et al., 2013; Wong et al., 2018). On average, most lncRNAs have low expression though in some tissues or at specific developmental stages of the disease, elevated expression of lncRNAs can be observed (Ward et al., 2015), indicating the regulatory or specific role of lncRNA in disease development.

#### LncRNAs in Hepatic Lipid Regulation, Steatosis and NASH

The role of lncRNAs in liver disease is still not fully understood. Previous reviews of lncRNAs in liver disease (Takahashi et al., 2014), fibrosis (Jiang and Zhang, 2017; Hanson et al., 2018) and HCC (Qiu et al., 2017) have proposed that lncRNAs play an important role in the pathogenesis of liver diseases. Among these known lncRNAs, H19 was the first to be discovered and associated with liver disease (Ariel et al., 1998). Following that, many other lncRNAs have been identified to be involved in liver disease, particularly in HCC progression, including MEG3 (Braconi et al., 2011a), HULC (Panzitt et al., 2007), MALAT-1 (Lai et al., 2012), HOTAIR (Geng et al., 2011), TUC338 (Braconi et al., 2011b) and others (Takahashi et al., 2014). Despite what has been reported, the exact regulatory roles of lncRNAs in liver disease and NAFLD pathogenesis are still unknown.

Long non-coding RNAs are shown to regulate hepatic lipid regulation and metabolism (Figure 1). A small study of NAFLD patients (*n* = 5) has profiled the lncRNAs expression in the liver tissues and found that 535 lncRNAs had increased expression, and 1200 lncRNAs had reduced expression (Sun et al., 2015). These dysregulated lncRNAs were further investigated *in silico* for their functional and regulatory roles, in which many of these lncRNAs are involved in small molecule metabolic processes and regulation of cytoplasmic and endoplasmic reticulum parts (Sun et al., 2015). Profiling of lncRNAs in animal models of NAFLD revealed more information about lncRNAs’ involvement in NAFLD. A study of NAFLD mice induced by high-fat diets showed that a total of 111 lncRNAs had increased expression, and 180 lncRNAs had reduced expression compared to the normal controls (Chen et al., 2017b). In this study (Chen et al., 2017b), seven lncRNAs were identified to be involved in the regulation of circadian rhythm. Upregulation of the lncRNA FLRL6 expression was shown to positively regulate *PER2* expression, a regulator of circadian rhythm (Chen et al., 2017b). Participation of lncRNA FLRL6 in the regulation of *PER2* expression may be important in NAFLD progression, as *PER2* can regulate hepatic lipid metabolism via PPARγ (Grimaldi et al., 2010). Another identified lncRNA is FLRL2, in which its expression was reduced in NAFLD mice and the expression of its protein-coding gene, *ARNT1*, was also reduced (Chen et al., 2017b). Reduced *ARNT1* expression was implicated in those with obesity (Paschos et al., 2012), high level of circulating fatty acids and ectopic fat formation in the liver (Shimba et al., 2011) suggesting there is a possible role of FLRL2 in hepatic lipid accumulation. Apart from that, two other lncRNAs were also identified to be involved in NAFLD namely, lncSTR that was shown to regulate systemic lipid metabolism (Li et al., 2015) and lncARS that was shown to regulate the fatty acid synthesis and oxidation via Akt/SREBP-1c pathway (Zhang et al., 2018). Identification of these dysregulated lncRNAs in hepatic lipid regulation may suggest that the expression of these lncRNAs can be used to identify the early stage of NAFLD.

#### LncRNAs in Hepatic Fibrosis and Injury

In terms of hepatic fibrosis, a recent and larger study of lncRNAs profiling in NASH patients (*n* = 48) identified a liver-specific pro-fibrotic lncRNA (lnc18q22.2) which was highly expressed in NASH patients, and this lncRNA expression was associated with greater NASH/NAFLD score and liver fibrosis index (Atanasovska et al., 2017). Similarly, another study of lncRNA profiling in the liver biopsies of NAFLD patients (*n* = 24) identified MALAT-1 as the potential regulator of inflammation and fibrosis (Leti et al., 2017). Investigation of hepatic tissue expression among fibrosis patients discovered more lncRNAs that were associated with fibrosis such as the HOXA distal transcript antisense RNA (HOTTIP) lncRNA that was elevated in fibrosis patients and even more so in cirrhosis patients (Li et al., 2018), thus implying that HOTTIP expression may indeed represent hepatic injury severity. Another reported lncRNA is linc-SCRG1 that was upregulated in human cirrhotic tissues and its inhibition led to a reduction of fibrosis-related genes and apoptosis (Wu et al., 2019). The mechanism on how linc-SCRG1 promotes fibrosis was shown to be via the inhibition of tristetraprolin (*TTP*) expression, which is a RNA-binding protein that is involved in the degradation of proteins (Wu et al., 2019).

Apart from human studies, animal models of liver fibrosis or injury also revealed more pro-fibrotic lncRNAs. The genome-wide profiling in animal models of liver fibrosis found a liver fibrosis-associated lncRNA1 (lnc-LFAR1) that was upregulated in HSCs and its direct binding to Smad2/3 proteins promoted TGFβ and Notch pathway activation and hepatocyte apoptosis (Zhang et al., 2017). Besides, a few other lncRNAs were also identified including the Alu-Mediated p21 Transcriptional Regulator (APTR) (Yu et al., 2015b), Plasmacytoma Variant Translocation 1 (PVT1) (Zheng et al., 2016), Homeobox (HOX) Transcript Antisense RNA (HOTAIR) (Bian et al., 2017), LncRNA H19 (Zhu et al.) and Nuclear Enriched Abundant Transcript 1 (NEAT1) (Yu et al., 2017b), in which all of these lncRNAs were upregulated in fibrotic tissues and activated HSCs. Most of these lncRNAs exerted their effects via ACTA2 and collagen, type 1, alpha 1 (COL1A1) proteins. With the identification of these pro-fibrotic lncRNAs, they can be used as biomarkers for fibrosis progression and the inhibition of their expression can be utilized as a treatment strategy.

As for lncRNAs with an anti-fibrotic role in the liver, genome-wide lncRNAs profiling in the rat model of liver fibrosis had identified 231 differentially expressed lncRNAs and characterized one lncRNA, namely NR_002155.1 that was able to suppress HSCs activation as it was significantly reduced in fibrotic liver (Gong et al., 2018). Other studies have also identified three other lncRNAs such as, MEG3 (Yu et al., 2018), GM5091 (Zhou B. et al., 2018) and lincRNA-p21 (Zheng et al., 2015), in which their expressions were reduced in fibrotic animals. Restoration of MEG3 and GM5091 expression caused down-regulation of *α-SMA* and *COL1A1* expression (Yu et al., 2018; Zhou B. et al., 2018). Similar to miRNAs and other conventional biomarkers, high false-positive and false-negative detections were also observed in these lncRNAs studies. Further research is required to fully characterize these lncRNAs particularly about their functional targets and their secretion into circulation.

#### Co-regulatory Network Between lncRNAs and miRNAs

One of the important findings in lncRNA studies is the role of lncRNA as a miRNA sponge and can prevent their actions toward the target mRNAs. This co-regulatory network between lncRNA and miRNA may indeed unravel the underlying mechanisms of NAFLD pathogenesis, though there are limited evidence available to support this molecular network (Figure 1). In the regulation of hepatic lipid, one study showed that lncRNA, NEAT1 increased adipose triglyceride lipase (*ATGL*) expression by competitive binding to miR124-3p and consequently increased levels of diacylglycerol and FFAs (Liu et al., 2018). This interaction between NEAT1/miR-124-3p disrupted the lipolysis and increased fatty acid oxidation via PPARα signaling in liver cells (Liu et al., 2018). Another study also demonstrated that miR-136 could suppress MEG3 and nuclear factor erythroid 2-related factor 2 (NRF2) expression and causing increased of TG contents and hepatocytes deaths (Wang and Wang, 2018). Due to these limited findings on the co-regulatory network between lncRNAs and miRNAs to control hepatic lipid regulation and metabolism, further work is needed to confirm these previous findings.

In contrast, there are more studies done to investigate the co-regulatory network between lncRNA and other molecules in hepatic liver fibrosis or injury. One such interaction is the relationship between lncRNA, NEAT1 and miR-122 in the regulation of liver fibrosis (Yu et al., 2017b). In this animal model of fibrosis (Yu et al., 2017b), it was demonstrated that miR-122 reduced the Kruppel-like factor 6 (KLF6) expression and NEAT1 inhibited the miR-122 regulatory action via competitive binding to miR-122. Suppression of miR-122 by NEAT1 caused increased of *KLF6* expression in HSCs and promoted fibrogenesis (Yu et al., 2017b), thus confirming the co-regulatory network of NEAT1/miR-122/KLF6 signaling cascade in fibrosis. Similarly, in other studies of fibrotic animals and HSCs, other co-regulatory networks were identified including, the HOTAIR/miR-29b/PTEN cascade (Yu et al., 2017a), linc-p21/miR-181b/PTEN cascade (Yu et al., 2016), PVT1/miR-152/PTCH1 signaling (Zheng et al., 2016), H19/miR-148a/USP4 and TGF-β pathway (Zhu J. et al., 2018), MALAT1/miR-101b/RAS-related C3 botulinum substrate 1 (RAC1) (Yu et al., 2015a), HOTTIP/miR-148a/TGF-β receptors (Li et al., 2018), HOTAIR/miR-148b/DNMT1 (Bian et al., 2017) and MEG3/miR-212/Smoothened (SMO) signaling (Yu et al., 2018). Interestingly, in one study of fibrotic animals, lncRNA HOTAIR mediated suppression of another lncRNA, MEG3 via epigenetic mechanisms (Bian et al., 2017). It was shown that HOTAIR acted as a platform or guide for binding of polycomb repressive complex 2 (PRC2) to the promoter region of MEG3 gene and induced histone H3K27me3 suppression (Bian et al., 2017). This promoter modifications then led to an inhibition of MEG3 expression and promoted liver fibrosis (Bian et al., 2017). This evidence of lncRNA-lncRNA interaction adds more complexity to the known molecular regulatory network, especially in liver fibrosis.

### Circular RNAs (circRNAs)

Circular RNAs (circRNAs) are the structurally covalent loop RNAs without a 5’-cap and 3’-tail (Yao et al., 2017). Previous studies have shown that circRNAs are the products of back-splicing events, which allows the formation of the loop structure (Wilusz Jeremy, 2018). This circular structure provides greater stability for this RNA species due to the protection against degradation by the exoribonucleases. With advancement in RNA sequencing analysis and in-depth molecular research, circRNAs are shown to regulate the gene expression at transcription and post-transcription levels by acting as a miRNA sponge as well as interacting with lncRNAs, mRNA and proteins (Wilusz Jeremy, 2018). Research in circRNAs has garnered much interest recently due to the circRNAs stability in the circulation and their potential to serve as biomarkers or therapeutic targets (Abu and Jamal, 2016; Wilusz Jeremy, 2018). Majority of the current studies on circRNAs in liver diseases are focused on HCC and hepatitis (Yao et al., 2017), with limited information available for NAFLD development and progression. Due to this limitation, we discussed the circRNAs role in NAFLD by exploring the co-regulatory network between circRNAs and their miRNA targets as well as their downstream molecular targets (Figure 1).

#### CircRNAs in Hepatic Lipid Regulation, Steatosis and NASH

The regulatory network between circRNAs and miRNAs to control gene expression has emerged as a new understanding of the molecular regulation in disease development (Figure 1). A study performed by Jin et al. (2016) profiled circRNAs expression from liver tissues of NASH mice and they found that 69 circRNAs had increased expression, and 63 circRNAs had reduced expression (Jin et al., 2016). Among these circRNAs, three of them have potential interaction with miR-122 as its sponge, thus affecting their respective genes in NASH (Jin et al., 2016). These include circRNA_002581 interaction with miR-122 and its respective gene of *SLC1A5*, circRNA_002581 interaction with miR-122 and its respective gene *PLP2* and circRNA_002581 interaction with miR-122 and its respective gene, *CPEB1* (Jin et al., 2016). Identification of this circRNA/miRNA/mRNA interaction thus establishes the role of circRNAs in NASH and possibly in NAFLD.

Another exploratory circRNAs profiling experiment was performed in HepG2 cells with hepatic steatosis induced by a high-fat stimulation (Guo et al., 2017b). A total of 357 circRNAs were found to be dysregulated with a reduced expression of circRNA_021412 and confirmation of co-regulation of *LPIN1* expression by circRNA_021412 and miR-1972 (Guo et al., 2017b). Another study pursued an in-depth molecular analysis of circRNA_0046367 expression and function in NAFLD. Expression of circRNA_0046367 was reduced in hepatic steatosis resulting in an increased expression of miR-34a (Guo et al., 2017a). miR-34a was shown to reduce *PPARα* expression, and the presence of circRNA_0046367 inhibited this miR-34a suppression of *PPARα* expression, in conjunction with evidence of improved mitochondrial function and prevention of hepatotoxicity (Guo et al., 2017a). This co-regulation of *PPARα* expression by circRNA and miR-34a was replicated again by the same researchers but using a different circRNA, circRNA_0046366 that also showed a prevention of hepatic steatosis (Guo et al., 2018). Although only a few studies have embarked on circRNAs research in NAFLD, this identification and the proof-of-concept study to elucidate the co-regulatory network of circRNA-miRNA involved in hepatic lipid regulation, have opened a new window of understanding the molecular regulation underlying the early stage of NAFLD pathogenesis.

#### CircRNAs in Hepatic Fibrosis and Cirrhosis

Studies of circRNAs in hepatic fibrosis have identified few circRNAs that are associated with fibrosis or liver injury. A microarray profiling of fibrotic HSCs induced by irradiation showed that 179 circRNAs expression were upregulated and 630 circRNAs expression were downregulated (Chen et al., 2017c). In this study (Chen et al., 2017c), they also investigated the role of circ_0071410 in HSCs activation and found that circ_0074410 reduced miR-9-5p expression and promoted HSC activation via α-SMA protein. Similarly, a global microarray profiling of circRNAs in the animal model of fibrosis revealed that 69 circRNAs were differentially expressed in the fibrotic liver tissues, with 14 of them had increased expression, and 55 had reduced expression (Zhou Y. et al., 2018). Among these circRNAs, circRNA_34116 was shown to inhibit HSCs activation possibly via miR-22-3p and its targets, BMP7 as predicted by the bioinformatics analysis (Zhou Y. et al., 2018). Another study of HSC inhibition by thymosin beta 4 treatment has identified 644 circRNAs that were differentially expressed and circRNA_0067835 expression was significantly increased in activated HSCs (Zhu L. et al., 2018). It was also shown that circRNA_0067835 acted as a miR-155 sponge and elevated the *FOXO3a* expression thus promoted HSCs activation and fibrosis (Zhu L. et al., 2018). Despite these limited findings of circRNAs roles in liver fibrosis, the evidence of their regulatory interaction with miRNAs and their target mRNAs showed that understanding the molecular regulation and mechanisms underlying liver fibrosis could potentially discover a novel therapeutic target.

### Other Emerging Regulatory ncRNAs

Besides the well-established ncRNAs, the P-element-induced wimpy testis (PIWI)-interacting RNAs (piRNAs) has garnered new interest as the new small regulatory ncRNAs. The piRNAs are similar in length to miRNAs (∼26–30 nucleotides) and have the 2’-O-methylation at the 3’-end (Weng et al., 2019). Unlike miRNAs, piRNAs precursors are single-stranded transcripts without any secondary structures and these precursors are generated from the specific genomic regions with repetitive elements (Weng et al., 2019). The precursors piRNAs are then modified post-transcriptionally (2’-O-methylation) to produce mature piRNAs and loaded onto PIWI family proteins to form silencing complexes to repress transposable elements (TEs), at both transcriptional and post-transcriptional levels in germline cells (Iwasaki et al., 2015; Sarkar et al., 2017). The presence of TEs in the human genome is high with up to 50% from overall genome components. However, the majority of these TEs are silenced (International Human Genome Sequencing Consortium, 2001; de Koning et al., 2011; Wang and Jordan, 2018) to prevent their notorious effects of causing genome instability via chromosomal breakages, insertions and genomic rearrangements (Wang and Jordan, 2018). Thus, piRNA is known as the protector of genome integrity (Iwasaki et al., 2015; Sarkar et al., 2017) due to their suppression of TEs. Previous studies showed that piRNAs can regulate mRNA and lncRNAs expression, in which piRNAs are implicated in mitochondrial surface lipid signaling (Huang et al., 2011; Shiromoto et al., 2013). However, to which extent these piRNAs are involved in NAFLD development and progression remain unknown, thus requires further investigation to evaluate these piRNAs in the regulation of lipid metabolism and signaling.

Another ncRNA that has been on the spotlight recently is the enhancer RNAs (eRNAs) (De Santa et al., 2010; Kim et al., 2010; Djebali et al., 2012) that usually are 0.5–5 kb in size and act as a transcription regulator via the enhancer molecules (Chen et al., 2017a; Azofeifa et al., 2018). The exact mechanism by which the eRNAs regulate the gene expression is still unknown. Studies have shown that eRNAs behave similarly to lncRNAs, in which they both regulate or promote the expression of nearby genes (Li et al., 2013; Melo et al., 2013), mainly via the promoter or enhancer regions (Bose and Berger, 2017; Meng and Bartholomew, 2017; Jiao et al., 2018). Limited information is available on the regulatory role of eRNAs in disease and much less for NAFLD. Interestingly, a recent study showed that eRNAs with m^5^C mark were associated with metabolic stress *in vitro* and *in vivo*; specifically, these eRNAs interacted with *PGC1α* to regulate *NSUN7* and *SIRT1* gene expression (Aguilo et al., 2016). This finding suggests that eRNAs may be involved in NAFLD progression, as both *PGC1α* and *SIRT1* are implicated in fatty acid oxidation and NAFLD development (Rodgers et al., 2005; Bellanti et al., 2014; Choi et al., 2017).

## Regulatory ncRNAs: Challenges and Future Direction

Among the ncRNAs in NAFLD, miRNAs is the most well-defined and have been extensively studied (Baffy, 2015; Gerhard and DiStefano, 2015; DiStefano and Gerhard, 2016; He et al., 2016; Otsuka et al., 2016; Lin et al., 2017). Emerging reports on other regulatory ncRNAs, including lncRNAs, circRNAs, piRNAs, and eRNAs have highlighted that co-regulation and interactions between these ncRNAs may expand the current understanding of the molecular regulation and its complexity in disease development and progression. The co-regulatory networks of circRNAs, lncRNAs, and miRNAs in NAFLD pathophysiology offer new possibilities in finding the biomarkers and therapeutic targets. Identification of non-invasive and sensitive biomarkers for NASH and fibrosis is crucial as the current liver biopsy method to assess NASH/fibrosis can cause further complication to liver health and the imaging methods can only detect steatosis but not NASH (Wruck et al., 2017). To date, many of these studies remain in preliminary stages with most of the works are done focusing on profiling and identifying their expressions in correlation to the NAFLD stages. More studies are needed to elucidate the mechanisms of how these ncRNAs regulate NAFLD progression and which molecules they interact with, especially in regards to lncRNAs that have diverse biological functions (Takahashi et al., 2014). Unlike miRNAs, lncRNAs are poorly conserved between species, thus to experimentally demonstrate their functions in *in vivo* studies is challenging. Similarly, research of circRNAs in NAFLD is still in its infancy but their ability to control miRNAs and their stability in the circulating biofluids are making them as the most promising biomarker and therapeutic agent. Therefore, in-depth understanding of these ncRNAs in NAFLD pathogenesis and their efficiency and specificity as biomarkers or therapeutic targets are the unmet need for better NAFLD outcomes and disease management.

## Author Contributions

SAS drafted and wrote the manuscript. NM wrote the manuscript and critically reviewed the manuscript. RJ critically advised and reviewed the manuscript.

## Conflict of Interest Statement

The authors declare that the research was conducted in the absence of any commercial or financial relationships that could be construed as a potential conflict of interest.
